# Enzymatic Preparation and Characterization of Spherical Microparticles Composed of Artificial Lignin and TEMPO-Oxidized Cellulose Nanofiber

**DOI:** 10.3390/nano11040917

**Published:** 2021-04-03

**Authors:** Naoya Fukuda, Mayumi Hatakeyama, Takuya Kitaoka

**Affiliations:** Department of Agro-Environmental Sciences, Graduate School of Bioresource and Bioenvironmental Sciences, Kyushu University, Fukuoka 819-0395, Japan; f.naoya708@agr.kyushu-u.ac.jp (N.F.); m_hatakeyama@agr.kyushu-u.ac.jp (M.H.)

**Keywords:** dehydrogenative polymer, enzymatic radical coupling, lignin, nanocellulose, microsphere, TEMPO-oxidized cellulose nanofiber

## Abstract

A one-pot and one-step enzymatic synthesis of submicron-order spherical microparticles composed of dehydrogenative polymers (DHPs) of coniferyl alcohol as a typical lignin precursor and TEMPO-oxidized cellulose nanofibers (TOCNFs) was investigated. Horseradish peroxidase enzymatically catalyzed the radical coupling of coniferyl alcohol in an aqueous suspension of TOCNFs, resulting in the formation of spherical microparticles with a diameter and sphericity index of approximately 0.8 μm and 0.95, respectively. The ζ-potential of TOCNF-functionalized DHP microspheres was about −40 mV, indicating that the colloidal systems had good stability. Nanofibrous components were clearly observed on the microparticle surface by scanning electron microscopy, while some TOCNFs were confirmed to be inside the microparticles by confocal laser scanning microscopy with Calcofluor white staining. As both cellulose and lignin are natural polymers known to biodegrade, even in the sea, these woody TOCNF−DHP microparticle nanocomposites were expected to be promising alternatives to fossil resource-derived microbeads in cosmetic applications.

## 1. Introduction

The adverse impact of microplastics on marine ecosystems has become an urgent issue to solve [1,2,3,4,5]. In general, microplastics are small plastic debris derived from various nonbiodegradable polymer materials used in daily life [3]. Initially, the gradual downsizing of large polymer materials, such as plastic bags and bottles, to small fractions, known as secondary microplastics, was considered a major issue. However, recently, primary microplastics, such as microparticles (MPs) contained in facial cleaners and cosmetics, have resulted in more serious problems [4,5]. These microplastics flow out to the ocean and accumulate in the sea for a long time without any significant biodegradation. Therefore, primary small plastics must immediately be substituted with biodegradable alternatives, with many studies on enhancing the biodegradability of synthetic polymers having been conducted to develop environmentally friendly polymer microparticles [6,7,8,9].

Cellulose and lignin are two major components of wood cell walls [10,11]. Cellulose is a linear homopolymer composed only of β-D-glucopyranose, which forms crystalline microfibrils during biosynthesis owing to regular strong inter- and intra-molecular hydrogen bonds. The microfibrils in wood greatly contribute to its tough structure, endowing trees with rigidity [12,13,14]. Cellulose is well known as biodegradable, owing to enzymatic decomposition mediated by *endo*-/*exo*-cellulases and β-glucosidases secreted by fungi, bacteria, and microorganisms found in forests and the deep sea [15,16,17]. Meanwhile, lignin is an aromatic polymer formed via enzymatic radical coupling of several tautomeric isomers of phenylpropanoid monolignols [18]. Lignin in wood absorbs ultraviolet light and endows the wood architecture with hydrophobicity through accumulation between the cell walls, known as lignification [18,19]. Lignin is also enzymatically degraded by the action of white-rot fungi [20,21]. Furthermore, recent research has shown that deep-sea microorganisms, such as *Novosphingobium* strains, can break down lignin-specific structures [22]. Therefore, many studies have focused on developing sustainable, biodegradable, and ecofriendly materials from woody biomass for various applications [14,23,24,25].

In the last two decades, a new type of cellulose nanomaterial, cellulose nanofiber (CNF), has attracted much attention owing to its extraordinary nanoarchitectures and inherent crystalline fiber structure [14,23,24,26]. This novel nanomaterial is expected to have applications as a natural filler for reinforced plastics [27,28,29], food and cosmetic additive [30,31], flexible electronic devices [32,33], and in optoelectronics [34,35] owing to its fascinating physicochemical properties [14,23]. Recently, various MPs prepared by CNF-stabilized Pickering emulsion templating methods, in which thin CNF shells cover polymer cores, have received much interest regarding practical applications [36,37,38,39]. These CNF-integrated MPs have shown high pH-responsive properties [36], adsorption/desorption abilities for drug loading/release [37], and temperature-regulation capabilities [38]. Furthermore, CNF-stabilized MPs are expected to have cosmetic and medical applications. Fujisawa et al. reported using suspension polymerization to synthesize polystyrene and poly(divinylbenzene) MPs from corresponding monomers in an oil phase, stabilized by 2,2,6,6-tetramethylpiperidine 1-oxyl (TEMPO)-oxidized CNFs (TOCNFs) [36,37,39]. These TOCNF-covered MPs have unique core–shell structures, and TOCNFs decompose easily [16]. However, the polymer cores in the obtained MPs are not biodegradable, potentially resulting in marine pollution [1,5]. Therefore, biodegradable components are promising for use in the synthesis of MPs for cosmetic applications. In this context, cellulose and lignin are attractive natural resources expected to undergo microbial degradation in the deep sea. Therefore, the preparation of biodegradable MPs by combining CNF and lignin is a promising approach to the functional design of marine-degradable MPs.

**Figure 1:**
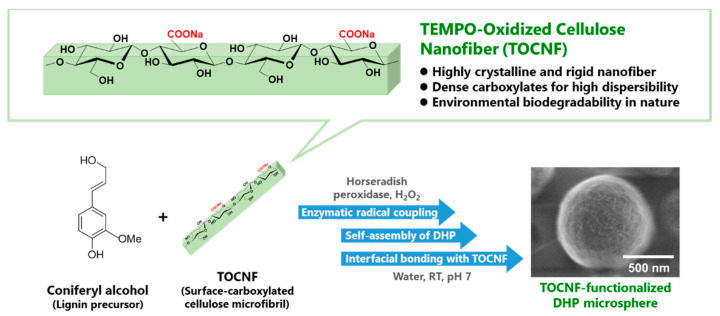
Schematic illustration of the research strategy: a one-pot and one-step green synthesis of spherical submicron-order TEMPO-oxidized cellulose nanofiber (TOCNF)-functionalized dehydrogenative polymer (DHP) microparticles.

In previous reports, lignin derivatives extracted from natural woods by various procedures have been used to fabricate MPs [40,41]. However, nature-derived lignin has crude and complex structures, which strongly affect its biodegradability and biocompatibility. On the other hand, to determine the biosynthesis mechanism of lignin in vivo, the in vitro synthesis of dehydrogenative polymers (DHPs) of coniferyl alcohol has long been investigated for use as an artificial lignin, enabling rough control of molecular structures [29,42,43]. Mićić et al. found that synthetic DHP molecules self-assemble to form fine particles [44,45]. Although several researchers have attempted to prepare MP composites using cellulose materials, few attempts to design spherical MPs combined with CNF have been reported because cellulose has a low affinity for DHPs, resulting in phase separation to form large aggregates [46,47]. Recently, our previous study revealed the affinity of CNF for DHP in the interfacial enzymatic synthesis of CNF−DHP nanocomposites [29]. In this article, we report the preliminary results of in situ enzymatic synthesis of DHPs from coniferyl alcohol in the presence of TOCNF without any organic solvents. TEMPO-based biomass conversion is expected as one of the promising green approaches to produce various nanomaterials such as CNFs isolated by TEMPO oxidation [48], chemo-enzymatically nanofibrillated lignocellulose [49], and biomass-derived chemicals by TEMPO-mediated catalysis [50]. In this work, nanoscale reconstruction of woody components was investigated to form cosmetic microspheres by using TOCNFs and DHPs, respectively, as surface-carboxylated cellulose microfibrils and artificial lignin (Figure 1). This one-pot green synthesis of spherical submicron-order TOCNF−DHP composite MPs is expected to provide new insight into the fabrication of marine-degradable microplastics.

## 2. Materials and Methods

### 2.1. Materials

TEMPO-oxidized CNF (TOCNF) used in this study was kindly provided by DKS Co. Ltd., Kyoto, Japan (RHEOCRYSTA, I-2SX, 2.3% (*w*/*v*), COONa = 1.55 mmol g^−1^ of TOCNF). Coniferyl alcohol, hydrogen peroxide (H_2_O_2_, 30% aqueous solution), and horseradish peroxidase (HRP) were purchased from Wako Pure Chemical Industries Ltd. (Osaka, Japan). Other chemicals and solvents were purchased from Sigma-Aldrich Japan, Ltd. (Tokyo, Japan), Wako Pure Chemical Industries, Ltd. (Osaka, Japan), and Tokyo Chemical Industry Co., Ltd. (Tokyo, Japan). All chemicals were used as received without further purification. Water used in this study was purified using a Barnstead Smart2Pure system (Thermo Scientific Co. Ltd., Tokyo, Japan).

### 2.2. Characterization of TOCNF

**Figure 2:**
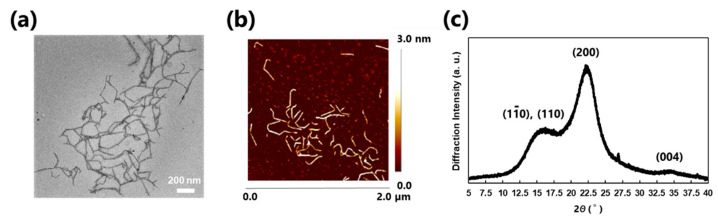
Characterization data of TOCNF used in this study. (**a**) transmission electron microscopy (TEM) image, (**b**) scanning probe microscopy (SPM) image (average height of each fiber = 2.1 ± 0.7 nm, *n* = 60 for each sample), and (**c**) X-ray diffraction (XRD) profile showing a typical cellulose I crystalline structure.

The nano-order morphology of TOCNF was observed using transmission electron microscopy (TEM; JEM-2100HCKM, JEOL Ltd., Tokyo, Japan) by staining with sodium phosphotungstate, and scanning probe microscopy (SPM; Dimension Icon, Bruker Japan Co. Ltd., Tokyo, Japan) equipped with a SCANASYST-AIR probe (*k* = 0.4 N m^−1^, *F*_0_ = 70 kHz) (Figure 2a,b, respectively). The crystalline structure of TOCNF was recorded by X-ray diffraction (XRD) analysis using a Rigaku SmartLab diffractometer (Rigaku Co., Tokyo, Japan) with Ni-filtered Cu Kα radiation (λ = 0.15418 nm) at 40 kV and 20 mA. The scanning rate was 0.5° min^−1^ with 0.05° intervals (Figure 2c). The carboxylate content of the TOCNF was determined by electrical conductivity titration [48]. The obtained data for TOCNF corresponded well with reported data [48]. TEM was performed at the Ultramicroscopy Research Center, Kyushu University. SPM and XRD analyses were conducted at the Center of Advanced Instrumental Analysis, Kyushu University.

### 2.3. Preparation of TOCNF-Functionalized DHP Microparticles

TOCNF–DHP MPs were enzymatically synthesized using the conventional DHP synthesis protocol, known as the Zulaufverfahren (ZL) method, i.e., “bulk” polymerization method [29,42]. Briefly, coniferyl alcohol (50 mg), 30% H_2_O_2_ (50 μL), and pure water (950 μL) were added to TOCNF suspension (0–1.0% (*w*/*v*), 4 mL). HRP solution (500 μL, 500 μg mL^−1^ in pure water) was then added to the reaction system. The reaction mixture was gently stirred at room temperature for 24 h to obtain TOCNF–DHP MPs, which were collected by centrifugation and thoroughly washed with purified water. The MP recovery ratios were up to 85% by weight. Another conventional DHP synthesis method, Zutropfverfahren (ZT; end-wise polymerization method) [29,42] was conducted as a control. The as-prepared MPs were observed using an optical microscope (DMI 4000B, Leica, Wetzlar, Germany) by mounting a droplet of the MP suspension on a glass slide. The ζ-potential of TOCNF−DHP MPs in an aqueous medium was measured at pH 7.0 using a Zetasizer Nano ZS instrument equipped with a 632-nm HeNe laser operating at a 173-degree detector angle (Malvern Panalytical, Tokyo, Japan). Fourier transform infrared (FTIR) spectroscopy was carried out to characterize as-prepared MPs using an FT/IR-620 spectrometer (JASCO, Tokyo, Japan). Each freeze-dried sample (ca. 2 mg) was mixed with 200 mg of KBr, followed by pressing it into transparent pellets. These samples were analyzed in the spectral range of 400–4000 cm^−1^ with a resolution of 2 cm^−1^.

### 2.4. Scanning Electron Microscopy (SEM)

MP suspension (1.0% (*w*/*v*), 3 μL) was dropped on a carbon tape and dried in a desiccator at room temperature for 24 h. These samples were then coated using an osmium coater (HPC-1SW, Vacuum Device, Ibaraki, Japan) for 1 s. SEM (SU8000, Hitachi High-Tech Co, Tokyo, Japan) analysis was performed at 0.7–1.0 kV. The diameter and sphericity index of MPs were manually calculated from the digital SEM images obtained. In brief, the orthogonal minor axis was divided by the major axis for 80 particles of each sample. The experiment was repeated twice to confirm the reproducibility. The cross-section of MP samples embedded in resin, cut using an ultramicrotome (Ultracut-UCT, Leica, Wetzlar, Germany), was observed by SEM analysis, performed at the Center of Advanced Instrumental Analysis, Kyushu University.

### 2.5. Confocal Laser Microscopy (CLSM)

MP suspension (1.0% (*w*/*v*), 2 μL) was mixed with 0.001% Calcofluor white stain solution (2 μL) on a glass slide, and the sample was then dried in the dark at room temperature for 24 h. The MPs were observed by confocal laser microscopy (TCS SP8 STED, Leica, Wetzlar, Germany) with excitation/emission at 405/415–500 nm, respectively. CLSM analysis was conducted at the Center for Advanced Instrumental and Educational Supports, Faculty of Agriculture, Kyushu University.

### 2.6. Drug Loading Test

Methylene blue (MB) and arginine (Arg) were used as model drugs for loading on MPs. MB and arginine hydrochloride were dissolved in phosphate buffer (pH 7.0, 10 mM) and sodium hydrogen carbonate aqueous solution (pH 8.4, 50 mM), respectively. The initial concentration of each model drug was adjusted to 5.0 mg L^−1^. Freeze-dried MPs (10 mg) were dispersed in the solution (1.0 mL) and stirred at room temperature for 2 h. The MP suspension was filtered through a 0.20-μm polytetrafluoroethylene membrane filter. The concentration of MB in the filtrate was determined using a UV–vis spectrophotometer (UH5300, Hitachi High-Tech Co., Tokyo, Japan) at the absorbance of MB (λ = 665 nm). The final Arg concentration in the solute was determined by reversed-phase high-performance liquid chromatography (RP-HPLC) analysis (Prominence UFLC, Shimadzu Co., Kyoto, Japan), by determining the peak intensity of dabsylated Arg [51]. Separation was achieved using a Shim-pack XR-ODS II column. Gradient elution mode with a flow rate of 1.0 mL min^−1^ was used, with 0.05% phosphate solution and acetonitrile used as mobile phases. The UV detector wavelength was set to 450 nm.

## 3. Results and Discussion

### 3.1. Preparation of Wood-Mimetic CNF–Lignin MPs

**Figure 3:**
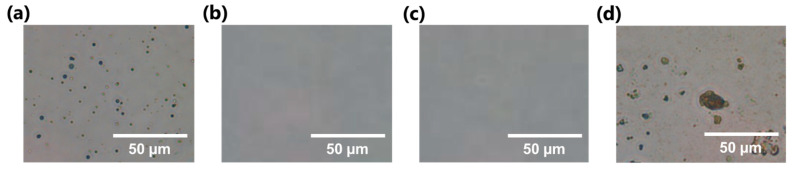
Optical images of DHP microparticles (MPs) synthesized (**a**) in the presence of 0.8% (*w*/*v*) TOCNF with HRP and H_2_O_2_, (**b**) w/o HRP, and (**c**) w/o H_2_O_2_ using the Zulaufverfahren (ZL) method. (**d**) Zutropfverfahren (ZT) method using 0.8% (*w*/*v*) TOCNF with HRP and H_2_O_2_.

DHPs were synthesized from coniferyl alcohol by HRP-mediated catalysis, namely the ZL method, in which all reactants are mixed together in one pot to generate radicals rapidly in 0.8% (*w*/*v*) TOCNF suspension. After washing MPs with pure water to remove excess TOCNF, spherical MPs were observed by optical microscopy (Figure 3a) [44,45]. In the absence of either HRP or H_2_O_2_, no MP formation occurred, as shown in Figure 3b,c, respectively. These results indicated that the MPs were enzymatically synthesized, and presumably composed of polymerized DHPs, but not precipitates of coniferyl alcohol monomer [29,42]. DHP MPs were also synthesized by the stepwise Zutropfverfahren (ZT) method, in which the radical generation rate is relatively slow [46]. ZT-type DHPs synthesized with TOCNF afforded irregular-shaped aggregates in addition to spherical MPs (Figure 3d). Sipponen et al. reported the successful fabrication of lignin nanoparticles by nanoprecipitation from aqueous ethanol, where initial precipitation of lignin formed small nuclei, followed by adsorption of other lignin components onto the as-formed nuclei, which resulted in nanoparticle growth [52]. When lignin precipitation occurs rapidly, numerous small nuclei are formed, eventually hindering the further growth of nanoparticles. In our study, the one-pot ZL method possibly promoted the formation of many small spherical precipitates in the presence of TOCNF, while slow generation of DHP precipitates by the ZT method caused gradual DHP deposition, resulting in the formation of relatively large nanoparticles. Furthermore, in the synthesis of ZT-type DHPs with TOCNF, coexisting TOCNF might disturb the smooth generation of DHPs, resulting in irregular-shaped aggregates. In contrast, the ZL method allowed the fabrication of spherical MPs through rapid radical formation and then self-assembly of the as-prepared DHPs, without inhibition by TOCNF. Morphological characterization data from SEM and CLSM analyses are presented in detail in the following section.

### 3.2. Morphological Characterization of TOCNF–DHP MPs

**Figure 4:**
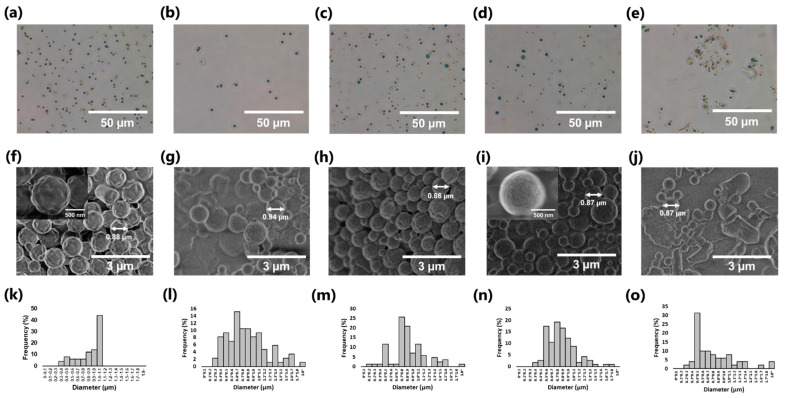
(**a**–**e**) Optical images, (**f**–**j**) SEM images, and (**k**–**o**) particle size distribution histograms of MPs synthesized with different concentrations of TOCNF: (**a**,**f**,**k**) 0% (*w*/*v*), (**b**,**g**,**l**) 0.3% (*w*/*v*), (**c**,**h**,**m**) 0.5% (*w*/*v*), (**d**,**i**,**n**) 0.8% (*w*/*v*), and (**e**,**j**,**o**) 1.0% (*w*/*v*). Insets of (**f**,**i**) show magnified picture of each one microparticle.

Optical microscopy images of MPs prepared using various concentrations of TOCNF are shown in Figure 4a–e. Spherical MPs were successfully synthesized with 0–0.8% (*w*/*v*) TOCNF suspension. Meanwhile, an irregular shape was observed in the case of 1.0% (*w*/*v*) TOCNF, owing to the higher TOCNF concentration affording a viscous suspension that inhibited DHP self-assembly [53]. The nanomorphology of as-prepared MPs was further investigated by SEM analysis (Figure 4f–j). As with optical microscopy results, TOCNF concentrations below 0.8% (*w*/*v*) resulted in formation of spherical MPs, possibly due to smooth DHP self-assembly. An increase in the TOCNF concentration provided MPs with fewer bumpy surfaces. Furthermore, nanofibrous components were observed on the MP surface, presumably indicating TOCNF fractions present on MP surfaces (inset of Figure 4i) [36,37], although TOCNF-free MP did not exhibit any similar components on the surface in the inset of Figure 4f. Therefore, our one-step green synthesis of wood-mimetic MPs enabled the formation of TOCNF-covered MPs containing DHP cores.

**Figure 5:**
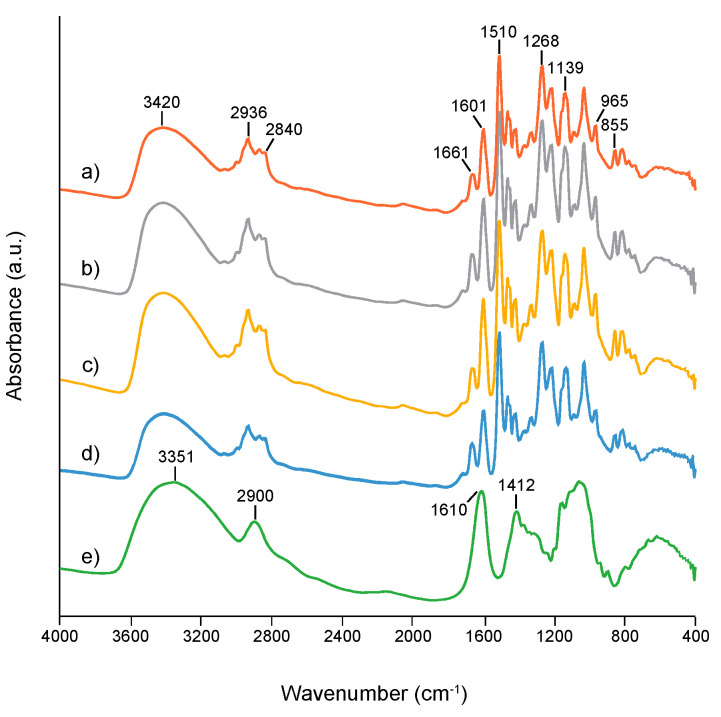
FTIR spectra of MPs synthesized with different concentrations of TOCNF: (**a**) 0% (*w*/*v*), (**b**) 0.3% (*w*/*v*), (**c**) 0.5% (*w*/*v*), (**d**) 0.8% (*w*/*v*), and (**e**) TOCNF used.

Spectroscopic characterization by FTIR analysis clearly revealed the successful formation of typical guaiacyl (G)-type DHP (Figure 5) [54]. A characteristic carbonyl peak at 1610 cm^−1^, originating from TOCNF [48], overlapped with the C=C stretching peak of G-rings at 1601 cm^−1^; however, the relative peak intensity at 1601/1510 for G-ring stretching of DHP increased from 0.487 to 0.544 with increasing amount of TOCNF, ranging from 0 to 0.5% (*w*/*v*). Therefore, as-prepared MPs were considered to contain the TOCNF components. Excess amount of TOCNF reduced the peak intensity around 1600 cm^−1^, possibly indicating the MP formation in an inappropriate fashion. Neither X-ray diffraction analysis nor X-ray photoelectron spectroscopy provided any significant peaks (data not shown), due to a slight amount of TOCNF on the MP surface below each detection limit.

The particle diameter and sphericity index of MPs were calculated from the SEM images (Figure 4f−i). The average diameter of MPs was about 0.8 μm, in the submicrometer (submicron) order, as listed in Table 1, which is expected to have practical cosmetic applications [55]. Furthermore, the standard deviation of each diameter slightly decreased as the TOCNF content increased from 0.3% (*w*/*v*) to 0.8% (*w*/*v*), while the diameter was unchanged. This was possibly due to TOCNF adsorbed on the MP surface acting like a Pickering emulsion stabilizer to improve MP stability in water [56]. The MP sphericity index was up to 0.94. True spheres are an important requirement of cosmetic use to improve cream spreadability of the foundation matrix [57]. The MP surface charges were determined by ζ-potential measurement (Table 1), showing −32.5 mV for CNF-free DHPs, which was consistent with the results of natural lignin nanoparticles [58]. The ζ-potential values of TOCNF–DHP MPs were up to about −40.0 mV, indicating negatively charged TOCNF adsorbed on MPs, which is expected to improve stability of the particle dispersion [59]. It has been reported that wood-derived TOCNFs have strong negative charges in the pH range of 5 to 7, equal to or greater than that at pH 7 [60]; therefore, as-prepared TOCNF–DHP MPs are expected to effectively use in a slightly acidic environment on the surfaces of human skins. Be that as it may, a long-term stability test is required to evaluate product applicability at practical levels.

**Figure 6:**
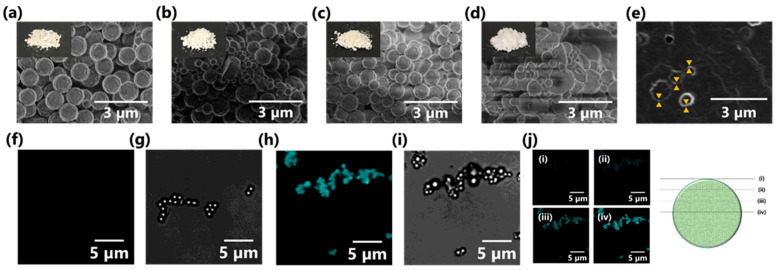
Morphology of freeze-dried MP powders prepared using different TOCNF concentrations: (**a**) 0% (*w*/*v*), (**b**) 0.3% (*w*/*v*), (**c**) 0.5% (*w*/*v*), and (**d**) 0.8% (*w*/*v*). Each inset corresponds to the optical image. (**e**) Cross-section SEM image of MPs from (**c**), with orange triangle markers indicating TOCNF-shell structures. CLSM images of MPs stained with Calcofluor white: (**f**,**g**) DHP MPs and (**h**−**j**) TOCNF–DHP MPs (TOCNF concentration, 0.5% (*w*/*v*)). (**f**,**h**,**j**) are fluorescent images. (**g**,**i**) are bright field images. Each panel of (**j**) is a Z-stacking image at each position, as illustrated.

Purified TOCNF–DHP MPs were freeze-dried to obtain solid MPs in powder form. In general, natural lignin is a dark brown color, which causes practical problems in cosmetic use [61]. However, the DHP MP powder had a creamy white color, as shown in the insets of Figure 6a–d. Very fine MPs were observed by SEM analysis (Figure 6a–d), but a high TOCNF concentration of 0.8% (*w*/*v*) seemed to induce morphological collapse of the MPs. This might be due to excess TOCNF on the MP surface aggregating during the freeze-drying process, resulting in some MP distortion. Herein, TOCNF–DHP MPs synthesized with 0.5% (*w*/*v*) TOCNF showed good shapes and were used in the following tests.

To evaluate the internal structure of the MPs, the MP cross-section was observed by SEM analysis. The sample embedded in resin was sliced using an ultramicrotome for SEM observation. The sliced image was not clear, but roughly exhibited furry TOCNF surrounding MPs, as shown in Figure 6e. This implied that TOCNF–DHP MPs had a shell layer of TOCNF [37]. CLSM analysis using Calcofluor white dye was conducted to visualize TOCNF inside the MPs (Figure 6f–i) [56]. TOCNF–DHP MPs showed strong fluorescence derived from Calcofluor white adsorbed to TOCNF (Figure 6h). In contrast, no significant fluorescence was observed in DHP MPs prepared without TOCNF (Figure 6f). These results indicated that fluorescent dye molecules selectively adsorbed to TOCNF. Furthermore, *Z*-stack imaging was conducted to show the interior distribution of TOCNF (Figure 6j). Each sliced image of the MPs was taken at several depth levels, ranging from the top to the middle of the MPs. In all pictures, fluorescence was detected over the whole MP cross-section. This suggested that TOCNF was embedded inside the MPs as well as on the surface. When DHPs were synthesized in the presence of TOCNF using the ZL method, some TOCNF was captured in the spherical MPs formed, possibly through hydrogen bonding and non-covalent π–interactions [62]. The TOCNFs adsorbed on the MP surface were stabilized by van der Waals interaction [63]. Covalent bond formation like lignin–carbohydrates complexes (LCCs) may be possible [64]. Accordingly, most TOCNF on the MPs formed a shell structure, while a significant amount of TOCNF was also embedded inside the MPs, although TOCNF inside the MPs was not observed in the SEM image (Figure 5e). CNF has high crystallinity and high mechanical strength, and has been widely used as a filler for reinforced plastics [27,28]. Therefore, incorporated TOCNF, exhibiting a rigid nanofiber form with the natural crystalline structure of cellulose I, as shown in Figure 2, would contribute to reinforcing the MPs when synthesized with TOCNF concentration of up to 0.5% (*w*/*v*). Moreover, different types of nanocellulose such as cellulose nanocrystals (CNCs) are also expected to use as a promising candidate for producing spherical CNC–DHP MPs, due to higher rigidity with more ordered alignments, possibly causing the better surface coverage and physical properties of MPs.

### 3.3. Preliminary Test for Drug-Loading on MPs

**Figure 7:**
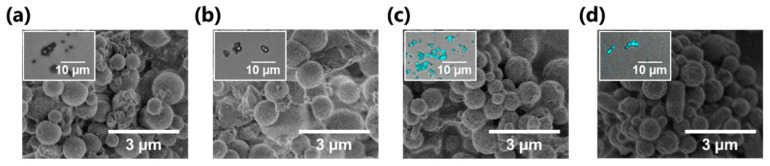
SEM and CLSM images (inset) of (**a**,**b**) DHP MPs and (**c**,**d**) TOCNF–DHP MPs (TOCNF, 0.5% (*w*/*v*)) after loading with (**a**,**c**) methylene blue (MB) and (**b**,**d**) arginine (Arg). CLSM images were obtained after Calcofluor white staining. Each inset is a merged picture of bright field and fluorescent images obtained by CLSM.

Cosmetic MPs require many practical performances such as long-term stability [65], biocompatibility [66], and UV-absorbing property [67]. Besides, drug-loading capability is one of the important factor for MP use in cosmetic applications [68]. In this study, two model compounds were subjected to adsorption behavior tests, namely, methylene blue (MB, a major model drug [37,69]) and arginine (Arg, a typical moisturizing ingredient for human skin [70]). MPs were poured into water solution containing each model drug, and stirred for 2 h. The MPs maintained their original spherical structures without any TOCNF peeling from the MP surface (Figure 7a–d). The drug loading was determined to measure the residual amount of each component in the supernatant. MB and Arg loadings of 94.9 ± 1.8% and 38.3 ± 0.2%, respectively, were obtained on the TOCNF–DHP MPs. These results indicated that TOCNF–DHP MPs had significant drug-loading capabilities. However, this preliminary test showed no specific adsorption to the TOCNF−DHP MPs, with more than 90% and 30% of MB and Arg, respectively, adsorbed to TOCNF-free DHP MPs. TOCNF contains many carboxylate groups on the nanofiber surface [48], and the reported core–shell MPs containing 2.5% (*w*/*v*) TOCNF on the surface have been reported to show good drug loading capability via electrostatic interaction between TOCNF and ionic drugs [37]. In this study, the TOCNF−DHP MPs possess relatively low amounts of TOCNF, resulting in little difference in the drug-loading capacity. Nonetheless, cellulose and lignin are expected to exhibit drug loading/release capabilities, as previously reported [37,58,69], indicating that the further design of TOCNF–DHP MP materials might enhance drug loading by tuning the combination of TOCNF and DHP in the enzymatic green synthesis without using organic solvents. The long-term stability test [65] still needs to be done for practical availability. In this work, there was no big difference in the morphology of the MPs before and after the drug loading test. Such stability test in cosmetic applications will be investigated in a future work. Our enzymatic preparation of hybrid MPs from DHP and TOCNF can be applied for other combination, e.g., ferulic acid and naturally-occurring phenols [71,72] with various nanocellulose. At this stage, it may involve the difficulty in the large-scale production; however, such a green approach would be highly expected further in the future to design various nanomaterials from biomass-based natural biomolecules.

## 4. Conclusions

The successful green synthesis of DHP MPs functionalized with TOCNF was achieved in a one-pot one-step aqueous process without any organic solvents. Horseradish peroxidase enzymatically catalyzed the radical coupling of coniferyl alcohol in an aqueous suspension of TOCNFs, and the resultant TOCNF–DHP composites possessed a spherical shape and submicron-order size of ca. 0.8 μm in diameter, which would be expected to have cosmetic applications. The TOCNF−DHP MP surfaces were covered with TOCNF that possessed ζ-potential of ca. −40 mV, possibly improving the stability of particle dispersion. Some TOCNF was also embedded inside the MPs. As both TOCNF and lignin are polymers known to be biodegradable in the sea, TOCNF−DHP MPs have great potential as alternatives to nonbiodegradable MPs.

## Figures and Tables

**Table 1:** Diameter, sphericity index, and ζ-potential of TOCNF–DHP MPs.

TOCNF Concentration (% (*w*/*v*))	Diameter (μm) ^1^	Sphericity ^1^	ζ-Potential (mV) ^2^
0.0	0.85 ± 0.24	0.97 ± 0.03	−32.5 ± 6.8
0.3	0.83 ± 0.36	0.95 ± 0.08	−39.8 ± 7.9
0.5	0.88 ± 0.31	0.94 ± 0.08	−40.7 ± 7.1
0.8	0.83 ± 0.26	0.95 ± 0.07	−38.4 ± 7.2
1.0	0.77 ± 0.49	0.80 ± 0.21	−40.4 ± 7.8

^1^ Calculated from SEM images at *n* = 80 for each sample. ^2^ Measured at *n* = 5 for each sample.

## Data Availability

Data presented in this study are available in this article.
